# Limb Preservation for Multiple Ray Osteomyelitis in Diabetic Forefoot Charcot: A Case Report

**DOI:** 10.1155/cro/1419433

**Published:** 2026-10-05

**Authors:** Kaissar Yammine, Marc Ghattas

**Affiliations:** ^1^ Diabetic Foot Clinic Department of Orthopedic Surgery Lebanese American University Medical Center-Rizk Hospital School of Medicine Lebanese American University Beyrouth Lebanon lau.edu.lb

**Keywords:** bone cement, Charcot neuroarthropathy, diabetic foot osteomyelitis, internal pedal amputation

## Abstract

This case report describes a very rare presentation of multifocal diabetic foot osteomyelitis (DFO) involving multiple rays and associated with forefoot Charcot neuroarthropathy. Based on magnetic resonance imaging findings and subsequent histopathological examination, the osteomyelitis was confirmed to be confined to the second and third metatarsals, the proximal phalanges of the hallux and second toe, and the metatarsal head and proximal phalangeal base of the fourth ray. Treatment consisted of complete resection of all infected bone in accordance with the principles of internal pedal amputation. The defects created following second‐ray metatarsectomy and proximal phalangectomy of the hallux were filled with antibiotic‐impregnated bone cement. At the 1‐year follow‐up, following this limb‐sparing procedure, the patient was pain‐free, free of infection, and able to bear full weight.

## 1. Introduction

Diabetic foot osteomyelitis (DFO) predominantly involve the forefoot, and in rare cases, it occurs in the absence of an ulcer with a clinical presentation of a single “sausage toe” [1]. In chronic cases, surgical management based on excision of the infected bone is indicated. Occasionally, a complete bone resection is required (phalangectomy and/or metatarsectomy), referred to as internal pedal amputation (IPA), and at times associated with antibiotic‐impregnated bone cement (AIBC) [2, 3]. On the other hand, diabetic Charcot foot disease rarely manifests in the forefoot [4].

This case report describes a very unusual presentation where two rare diabetic foot complications are combined. A multifocal osteomyelitis, presenting as three sausage toes extending to two metatarsals associated to a forefoot Charcot, treated successfully with multiple IPA combined and AIBC. This article follows the CARE guidelines for case reports [5].

## 2. Case Report

A 64‐year‐old man with a 15‐year history of Type 2 diabetes mellitus presented to our clinic with a swollen and painful right foot that had developed over the preceding 3 months following minor trauma, which resulted in a small puncture wound on the dorsal aspect of the midfoot. Two days after the injury, the foot became edematous and erythematous. A diagnosis of cellulitis was made at another institution, and the patient received 1 month of intravenous antibiotics, followed by 1 month of oral antibiotic therapy, without clinical improvement. The patient sought a second opinion at our clinic after being advised to undergo a transmetatarsal amputation.

Physical examination revealed a swollen, tense, and inflamed forefoot and midfoot with nonpitting edema. No skin ulceration or wound dehiscence was observed. The posterior tibial and dorsalis pedis pulses were palpable. The patient was febrile, with a body temperature of 38.6°C. Clinical examination and vascular ultrasonography confirmed adequate below‐knee arterial perfusion. Laboratory investigations demonstrated leukocytosis (13,400/mm^3^) and an elevated C‐reactive protein level (66 mg/L).

Plain radiographs demonstrated advanced osseous fragmentation involving the mid‐to‐distal shafts of the second (M2) and third (M3) metatarsals, as well as the bases of the second and fourth proximal phalanges, with associated surrounding dystrophic ossification. These findings initially suggested a diagnosis of forefoot Charcot neuroarthropathy. Magnetic resonance imaging (MRI) with gadolinium contrast demonstrated destructive osseous changes on T1‐weighted sequences, with contrast enhancement also involving the fourth metatarsal head and the proximal phalanx of the hallux (Figures 1, 2, and 3).

**Figure 1:**
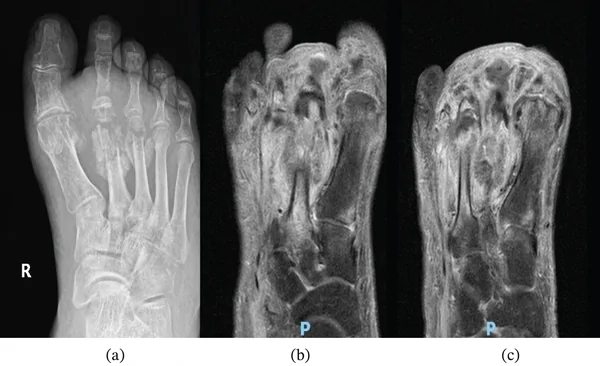
(a) X‐ray findings and (b,c) MRI T1 with contrast showing M2 and M3 infection.

**Figure 2:**
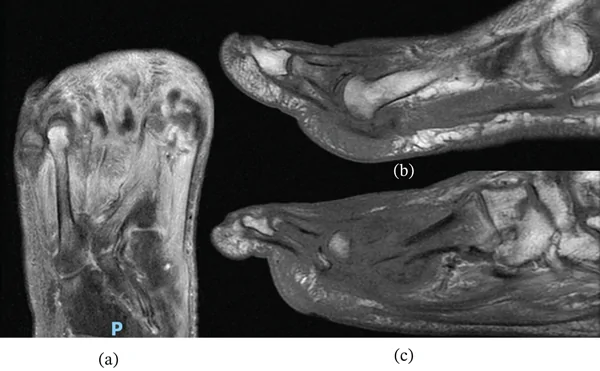
MRI T1 with contrast showing: (a) 4th metatarsal head infection, (b) proximal hallucal phalanx infection, and (c) proximal phalanx infection of the 4th toe.

**Figure 3:**
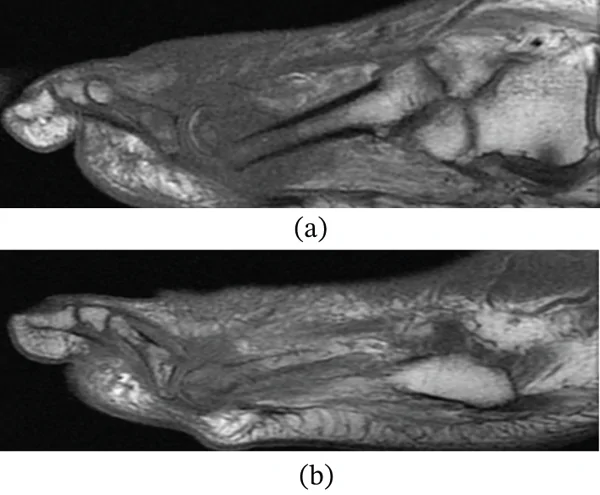
MRI T1 with contrast showing (a) M2 and (b) M3 infections.

A diagnosis of multifocal destructive DFO most probably coupled to a forefoot Charcot was established. A limb‐sparing procedure was therefore proposed, consisting of multiple‐ray IPAs, combined with AIBC to fill two resulting bone defects: the second metatarsal defect and the proximal phalangeal defect of the hallux.

### 2.1. Surgical Technique

Under general anesthesia combined with a peripheral nerve block, a thigh tourniquet was applied. The lower limb was then prepped and draped in sterile fashion. Four incisions were planned to achieve the procedure (Figure 4). First, a dorsal longitudinal incision centered over the second metatarsophalangeal (MTP) joint was performed. Intra‐articular purulence was identified. Complete phalangectomy of the proximal phalanx of the second toe was carried out. The second metatarsal head was released, and the shaft was progressively mobilized proximally using minimally invasive periosteal elevation. Second, a transverse dorsal incision over the second and third metatarsals allowed complete removal of M2 and proximal osteotomy of M3 using an oscillating saw. Third, a dorsal incision between the third and fourth toes enabled complete excision of M3. Resection arthroplasty of the fourth MTP joint was then performed, including resection of the distal M4 shaft and the base of the proximal phalanx. Fourth, a dorsal incision centered over the proximal phalanx of the hallux allowed complete excision of the involved bone. Figure 5 shows all the removed bone segments.

**Figure 4:**
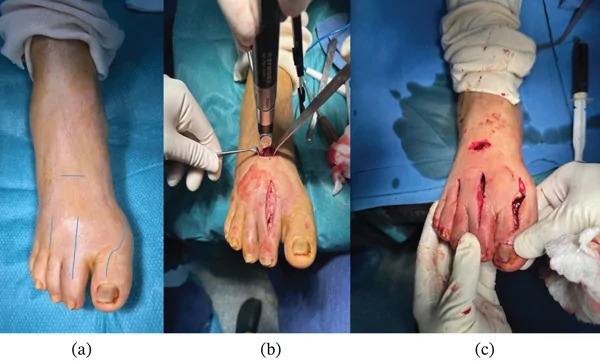
(a) Planned incisions, (b) osteotomy of the base of M2 and M3 through minimal incision, and (c) wound aspects after removing all infected bones.

**Figure 5:**
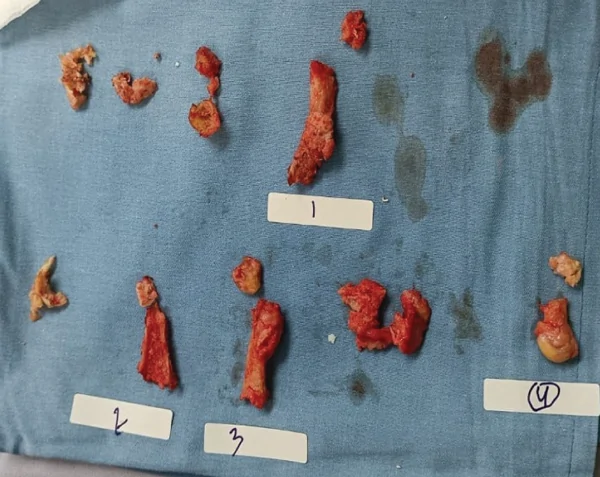
Display of the resected bones from each ray.

Following copious irrigation and meticulous hemostasis, antibiotic‐loaded bone cement was prepared and molded to fill the second ray and the hallux proximal phalanx defects. Cement preparation was based on polymethylmethacrylate (PMMA‐Gentafix, Teknimed, Germany) of 40 g mixed with 2 g of vancomycin. The cement was shaped to achieve an approximate volume ratio of 1:3 relative to the residual cavity, with the cement construct occupying roughly one‐third of the defect volume. Once the shape and volume were deemed satisfactory, the cement constructs were maintained on the surgical instruments throughout the polymerization process. After complete solidification, the cement elements were inserted into the postresection cavities (Figure 6).

**Figure 6:**
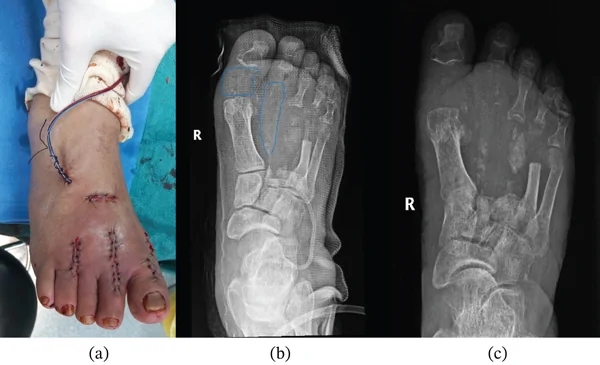
(a) Surgical immediate postoperative aspect, (b) immediate postoperative x‐ray (in blue the delimitation of the nonopaque cement), and (c) final x‐ray at 1 year.

Soft tissue specimens were sent for microbiological culture, and bone samples were submitted for histopathological analysis. A surgical drain was inserted, and the wounds were closed using nylon sutures. A forefoot off‐loading shoe was prescribed, and partial weight‐bearing was permitted. Broad‐spectrum antibiotic therapy was initiated postoperatively. Serial postoperative x‐rays were completed at Day 1, 1 month, 3 months, 6 months, and 12 months.

## 3. Results

Broad‐spectrum intravenous antibiotics (Carbapenem and Vancomycin) were initiated immediately until culture results. The drain was removed on postoperative Day 2. Apart from mild persistent edema, the postoperative course was uneventful. Clinical findings and inflammatory markers normalized within 13 days. Soft tissue cultures grew *Staphylococcus aureus* and *Proteus mirabilis*, and antibiotic therapy was subsequently adjusted (ciprofloxacin for 3 weeks) according to antimicrobial susceptibility testing.

Histopathological examination confirmed acute osteomyelitis with marked leukocytic infiltration involving all five medullary canals.

At 12‐month follow‐up, no recurrence of infection or wound‐related complications was observed. No radiological signs of Charcot progression or displaced cement were found on the 1‐year postoperative x‐ray (Figure 7). At the final follow‐up, the patient was pain‐free, with no clinical signs of infection or new deformity (Figure 8). However, no functional foot score was completed, the patient was fully weight‐bearing and highly satisfied with the functional and clinical outcome.

**Figure 7:**
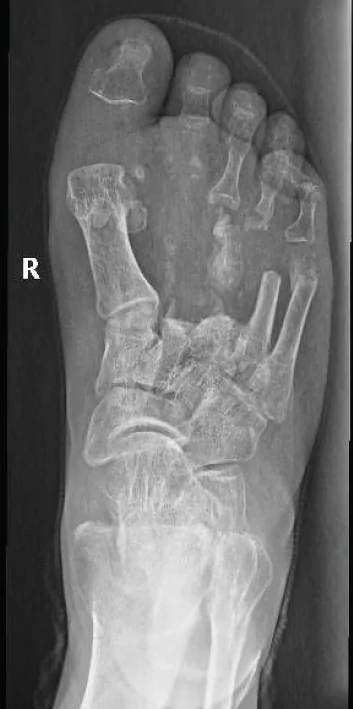
Final x‐rays at 12 months.

**Figure 8:**
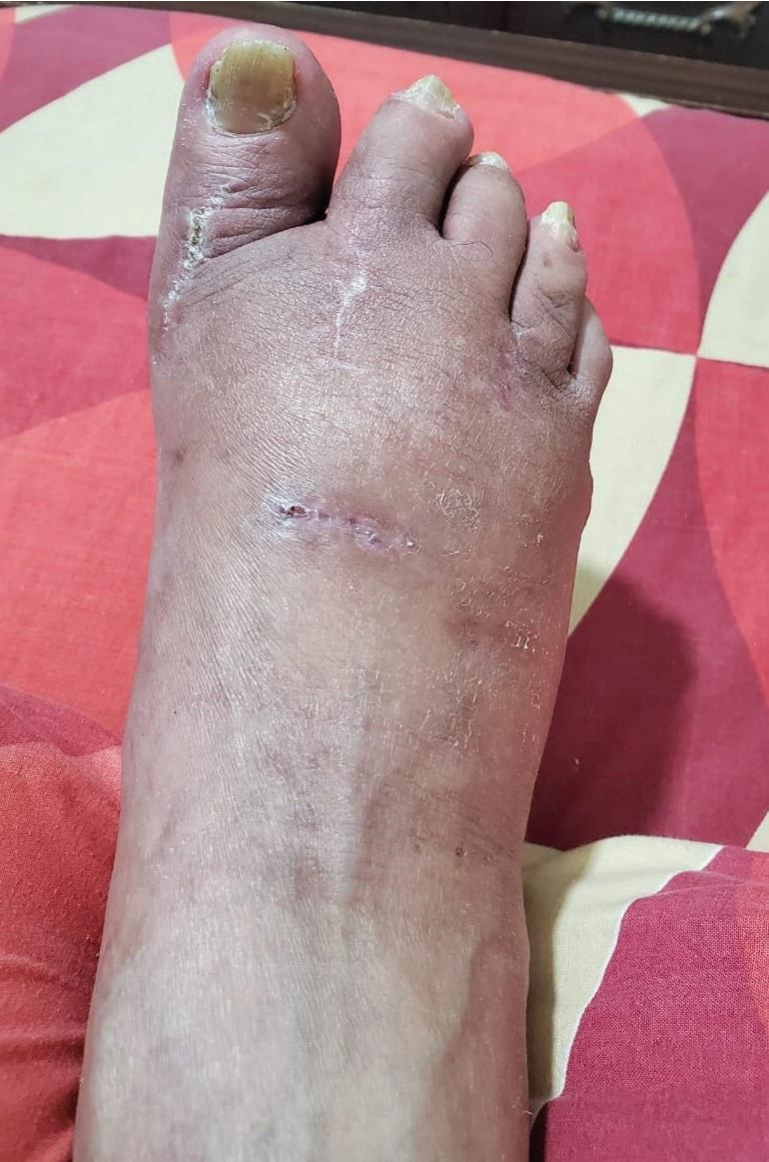
Final clinical photo.

## 4. Discussion

This case highlights the unlikely combination of two rare conditions, an acute forefoot Charcot associated with multiple ray osteomyelitis following a minor trauma and a puncture wound. It is well documented that diabetic neuropathy has a denervation effect on skeletal integrity; however, many triggers were reported to precipitate active Charcot syndrome where trauma is the most prevalent trigger, up to 36% [6, 7]. On the other hand, similarly to chronic wounds, puncture wounds of the foot in people with long‐term diabetes can result in severe infections and osteomyelitis [8].

Conservative management based on local wound care (when a DFU is present) and systemic antibiotic therapy is usually attempted initially in the treatment of DFO. However, long‐term success rates remain limited, and surgical intervention is frequently required [2]. When soft tissues are preserved, IPA was reported to be highly successful [1–3]. Though recent indications were typically reserved for single‐ray DFO, its use following complete resection of two metatarsals, two phalanges, and partial resection of an additional phalanx is highly unusual. Based on our limb‐sparing strategy when dealing with advanced diabetic foot complications, our assessment was that the residual presence of three metatarsals following excision of all affected bones could be biomechanically appropriate for a proper gait. Such initial evaluation coupled with properly conserved soft tissues weighted for multiple IPD rather than transmetatarsal amputation. In addition, the patient was an active physician, motivated and could acknowledge the rationale of the conservative procedure, where IPA would not hamper a later indication for a transmetatarsal amputation. The very few cases of forefoot Charcot published in the literature were mainly associated with myelopathy and other abnormalities affecting the spinal cord such as syringomyelia and spina bifida, and were all treated conservatively using off‐loading until the consolidation phase of the disease process [4, 9].

On the other hand, forefoot involvement in Charcot neuroarthropathy is also exceedingly rare, with an estimated rate between 2%–8% of diabetic Charcot cases [4]. Besides a history of recent foot surgery, minor trauma has been documented as a precipitating event to diabetic Charcot [10], as noted in our report too. In terms of differential diagnosis, the degree of metatarsal fragmentation (Eichenholtz Stage 1) appeared too advanced to be explained solely by infection within the 3‐month interval since the traumatic event and presentation. Classically, the radiological signs of acute/subacute osteomyelitis include regional osteopenia, bone erosion and periosteal reaction; intraosseous abscess (Brodie′s abscess) or sequestrum could be observed as well [11]. None of those signs were noted in our case.

Accurate determination of the extent of intramedullary infection is essential for achieving successful surgical outcomes. In previous studies, we reported the systematic use of MRI to determine the optimal level of metatarsal resection, which was typically approximately 1.5 cm proximal to the area of abnormal MRI signal intensity [2, 3]. When the abnormal signal extends proximally beyond a suitable level for limited resection, we do not hesitate to perform complete excision of the affected bone, thereby performing an IPA in order to minimize the risk of residual infection and subsequent recurrence.

Dead space formation, local infection control, and gait instability are three major concerns following complete resection of multiple metatarsals and phalanges. In this context, many reports justify the adjunctive use of AIBC as potential solutions to these challenges [2, 3, 12–14].

This case demonstrates that even in extreme presentations, amputation may still be avoided. Advanced multifocal DFO, particularly in the presence of preserved soft tissue viability, may benefit from aggressive limb‐sparing procedures.

## 5. Conclusion

This case report describes a very rare presentation of multiple‐ray DFO occurring in the setting of forefoot Charcot neuroarthropathy, successfully treated with multiple IPAs combined with AIBC. We believe that every reasonable effort should be made to preserve the limb in patients with advanced diabetic foot complications. Given the favorable outcomes associated with IPA and the established benefits of AIBC in the treatment of single‐ray DFO, extending this combined approach to multiple‐ray DFO appears both rational and justified.

## Funding

No funding was received for this manuscript.

## Ethics Statement

Ethical approval for this study was waived by the Institutional Review Board of our institution because this manuscript was based on a retrospective analysis of a patient case. However, written informed consent for the case publication and images was provided by the patient.

## Conflicts of Interest

The authors declare no conflicts of interest.

## Data Availability

The data that support the findings of this study are available from the corresponding author upon reasonable request.
